# Development and Evaluation of a HS-SPME GC-MS Method for Determining the Retention of Volatile Phenols by Cyclodextrin in Model Wine

**DOI:** 10.3390/molecules24193432

**Published:** 2019-09-21

**Authors:** Chao Dang, Kerry L. Wilkinson, Vladimir Jiranek, Dennis K. Taylor

**Affiliations:** 1 The University of Adelaide, School of Agriculture, Food and Wine, PMB 1, Glen Osmond, SA 5064, Australia; chao.dang@adelaide.edu.au (C.D.); vladimir.jiranek@adelaide.edu.au (V.J.); dennis.taylor@adelaide.edu.au (D.K.T.); 2 The Australian Research Council Training Centre for Innovative Wine Production, PMB 1, Glen Osmond, SA 5064, Australia

**Keywords:** *Brettanomyces*, cyclodextrins, gas chromatography-mass spectrometry, nuclear magnetic resonance, smoke taint, volatile phenols, wine

## Abstract

Volatile phenols exist in wine and can be markers for *Brettanomyces* and smoke taint off-odors. Cyclodextrins (CDs) are found to be capable of forming inclusion complexes with volatile phenols. Cross peaks on 2D ^1^H ROESY nuclear magnetic resonance (NMR) spectra demonstrated inclusion of volatile phenols in the β-CD cavity, while difference tests confirmed this resulted in a perceptible reduction of their sensory impact. However, a conventional headspace solid phase microextraction (HS-SPME) method using an isotopically labelled normalizing standard failed to quantify the residual volatile phenols by gas chromatography-mass spectrometry (GC-MS) because of inclusion of the standard by the CDs. A new method involving an additional liquid phase was developed and validated for quantitation of volatile phenols in the presence of CDs. The retention of eight volatile phenols by α-, β-, and γ-CD was subsequently studied.

## 1. Introduction

Volatile phenols are an important group of wine aroma compounds. Some volatile phenols, for example guaiacol, 4-methylguaiacol, vanillin, and eugenol, are routinely identified in wines aged in oak barrels, as a consequence of thermal degradation of lignin during the toasting process of cooperage [1]. These volatile phenols contribute to the smoky, vanilla and clove characters often associated with oak maturation [2,3]. However, volatile phenols are also responsible for certain off-odors in wine. *Brettanomyces* and/or *Dekkera* spoilage can result in the accumulation of 4-ethylguaiacol and 4-ethylphenol in wine, which at elevated concentrations can impart undesirable barnyard, sweaty, medicinal, and/or horsy notes [4]. Guaiacols, cresols, and syringols have been identified as markers of smoke taint, i.e., the objectionable smoky, ashy character observed in wines made from grapes exposed to bushfire smoke [5,6,7].

The wine industry has long sought strategies for mitigating off-odors, including those attributable to the presence of volatile phenols. Most amelioration strategies have involved the addition of sorptive materials such as yeast lees [8,9], activated carbon [10], and polyvinylpolypyrrolidone [11] to remove taint compounds from wine. However, these materials can also bind to volatiles responsible for desirable wine aromas and flavors. Reverse osmosis fractionation of wine prior to solid phase adsorption treatment has been used to achieve more selective removal of taint compounds [12,13]; while novel sorbents including esterified cellulose [14], polyaniline-based compounds [15] and molecularly imprinted polymers [16,17] have also been evaluated for the amelioration of taint because of the presence of volatile phenols in wine.

Cyclodextrins (CDs) are cyclic oligosaccharides comprising α-1,4-linked glucose units, the most common being α-CD, β-CD, and γ-CD, which comprise 6, 7, and 8 glucose units, respectively [18] (Figure S1). The spatial arrangement of sugars gives CDs a characteristic bucket shape, whereby the hydrophilic outer surface affords water solubility, while the hydrophobic inner cavity enables the formation of host-guest inclusion complexes with various molecules, including volatile phenols [18,19] (Figure S2). Numerous industries have exploited the encapsulation of volatile compounds by CDs, including those involved in the production of foods, beverages, and cosmetics, to stabilize, preserve, and/or mask aromas, flavors, and fragrances [20,21,22,23]. However, to date, there are few studies concerning the use of CDs in winemaking. The potential for β-CD to reduce the intensity of off-odors associated with *Brettanomyces* spoilage of red wine has been demonstrated [24]. β-CD has also been used to extract stilbenes, flavonols, and flavan-3-ols from grapes and pomace [25]. A key aim of the current study is therefore to determine to what extent volatile phenols associated with *Brettanomyces* spoilage and smoke taint can form inclusion complexes with CDs, so as to mitigate their impact on wine sensory properties. In order to achieve this aim, the concentration of volatile phenols should be determined before and after the addition of CD to wine. Headspace solid-phase microextraction (HS-SPME) has been shown to be a fast and effective sampling method for gas chromatography-mass spectrometry (GC-MS) analysis and it has been used extensively for the determination of volatile compounds in wine [26,27], including volatile phenols [28]. However, quantitative analysis relies on the addition of an appropriate normalizing standard, for example an isotopically labelled analogue in the case of stable isotope dilution assays [29], and the standards are equally subject to treatments on the sample mixture, such as the addition of CDs. Whereas conventional HS-SPME employs a three-phase extraction system, comprising the sample, its headspace, and the SPME fiber, in the current study, an additional liquid phase was introduced to overcome interactions between CDs and normalizing standards. This was achieved by inserting a glass ampoule containing the internal standard solution into the headspace vial, prior to analysis. This study describes the development and validation of a novel four-phase HS-SPME GC-MS method for determining the retention of volatile phenols by CD in model wine.

## 2. Results and Discussion

### 2.1. Traditional (Three-Phase) HS-SPME GC-MS, NMR, and Sensory Analysis

The conventional three-phase HS-SPME GC-MS method yielded excellent repeatability and linearity for quantification of guaiacol, 4-methylguaiacol, and 4-ethylphenol in model wine (Table S1). In the CD treatment assay, 25 g/L of α-CD, β-CD, and γ-CD were dissolved in the mixture before the internal standard was added. The relative peak area (RPA) of treatment groups and the controls were compared, but no significant differences in volatile phenol levels were observed for any of the CD treatments (Table 1). These results contradicted previous reports that β-CD significantly reduced the sensory perception of 4-ethylphenol in wine [24]. It was speculated that the CDs may have formed an inclusion complex with both the volatile phenols and the internal standards, equalizing changes in the relative response of both samples and standards following CD addition. In agreement with previously published work [27,28,29,30], the absolute peak areas were not reproducible, particularly when different fibers were used, and thus were not suitable for use in quantification.

**Figure 1:**
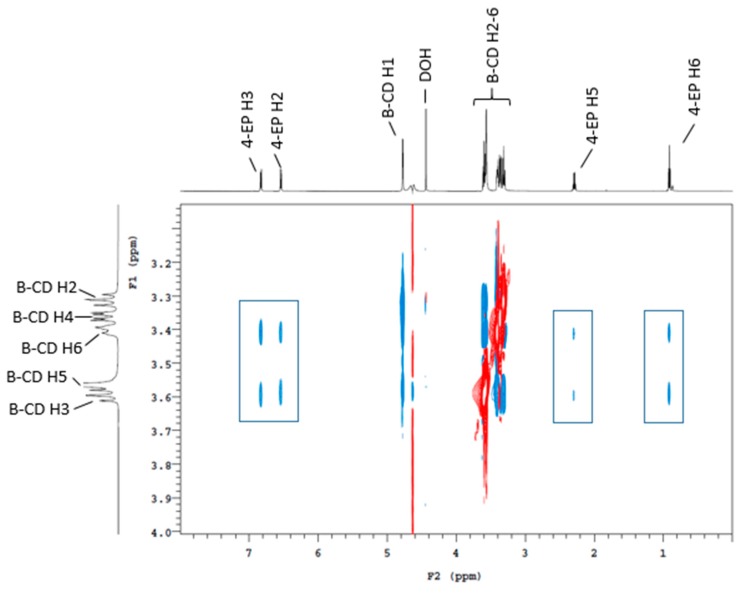
^1^H 2D ROESY NMR (600 MHz, pD 3.5 and 25 °C) spectrum of a D_2_O and d_5_-ethanol model wine containing 10^−3^ mol/L of 4-ethylphenol and 10^−2^ mol/L of β-CD. Rectangles indicate the cross-peaks arising from nuclear Overhauser effect (NOE) interactions between the annular H3, H5, and H6 protons of the CD and the aromatic and methyl protons of 4-ethylphenol.

To establish whether or not binding had occurred between the CDs and volatile phenols, NMR analyses were carried out on the mixture of β-CD and several volatile phenols (Figure 1). Cross peaks arising from the nuclear Overhauser effect (NOE) were observed between the protons in the β-CD cavity and volatile phenol protons, confirming the close spatial arrangement of these protons in an inclusion complex. This was further supported by sensory analysis, with 24 and 20 panelists (of 38) perceiving a difference in the “smoke taint” and “*Brettanomyces*” brackets, respectively, suggesting significant changes in the volatile phenol levels in the headspace after β-CD treatment. These results confirmed that the three-phase headspace SPME method, involving addition of the internal standard to the sample containing CDs, was not suitable for quantitative analysis.

### 2.2. Development of A Four-Phase HS-SPME GC-MS Method

In the current study, an ampoule comprising an additional liquid phase containing the internal standard was used to prevent CD interference. This ensured the standard could not be directly encapsulated by the CDs, but this modification significantly affected the kinetics of the existing SPME method. Conventionally, quantitative headspace SPME method is performed when a partition equilibrium of the target compound is achieved between three phases, namely the sample matrix, the headspace, and the fiber. The extraction of a given compound can then be expressed as:

C_0_*V_s_ = C_s_*V_s_ + C_h_*V_h_ + C_f_*V_f_ (1)

where C_0_ is the original concentration of the compound in the sample, C_s_ is the residual concentration remaining in the sample, Vs is the volume of the sample (liquid phase), C_h_ is the concentration of the compound in the headspace, V_h_ is the volume of the headspace (gas phase), C_f_ is the concentration of the compound on the SPME fiber coating, and V_f_ is the volume of the fiber (solid phase).

There are two equilibria in this process, i.e., the equilibrium between the sample and the headspace (K_1_) and that between the headspace and the fiber coating (K_2_). The equilibrium constants, i.e., Henry’s Law Constants, K_1_ and K_2_, are expressed as: K_1_ = C_s_/C_h_ and K_2_ = C_h_/C_f_, where the equilibration time is longer than ideal, good precision can be achieved, provided extraction conditions such as temperature, fiber penetration, and agitation are well controlled [31]. In the current study, the equilibrium is more complex, comprising distribution of volatile compounds in four phases during extraction, because of the presence of an additional liquid phase:

C_0_*V_s_ = C_s_*V_s_ + C_i_*V_i_ + C_h_*V_h_ + C_f_*V_f_ (2)

where C_i_ is the concentration of the internal standard in the additional liquid phase, and V_i_ is the volume of the additional liquid phase. An additional equilibrium constant, K_3_, exists for partitioning between the headspace and the additional liquid phase: K_3_ = C_h_/C_i_.

In the current study, it is hypothesized that the sample mixture containing the CDs would not meaningfully interfere with the volatile phenols present in the headspace because of the relatively short extraction time, so C_i_*V_i_ was considered to be zero. The method development employed in this study did not focus on modelling the overall process, but rather the practicality of the process in determining the retention of volatile phenols by CDs.

#### 2.2.1. Influence of Agitation

**Figure 2:**
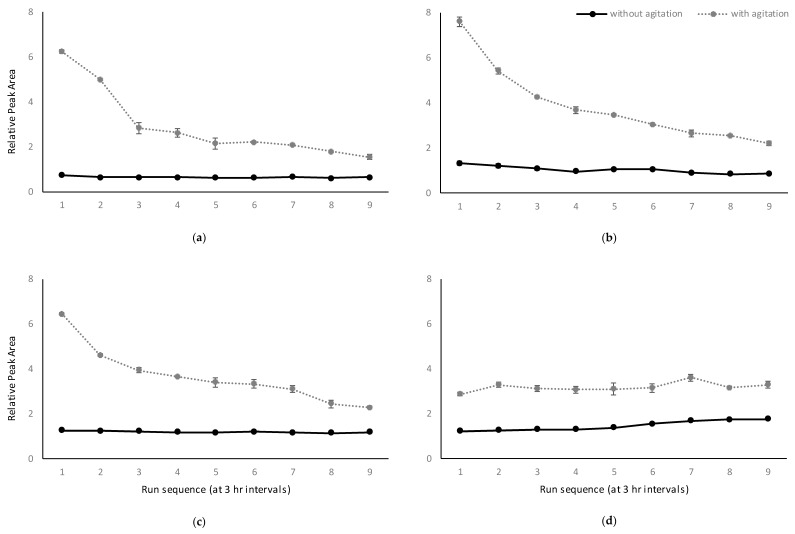
Effect of agitation during equilibration and internal standard volume, (**a**) 2 mL, (**b**) 1 mL, (**c**) 0.5 mL, and (**d**) 0.1 mL, on the relative peak area of guaiacol over time (i.e., expressed as run sequence at 3-hr intervals after the SPME vial was capped). Values are means of three replicates ± standard error.

The RPAs obtained for some samples changed significantly with time, before more constant levels were achieved. With the same volume of internal standard, agitated samples yielded significantly higher RPAs than non-agitated samples, particularly for lower equilibration times (Figure 2). To further investigate, absolute peak areas for *m*/*z* 124 (guaiacol) and *m*/*z* 127 (*d_3_*-guaiacol) were compared. Despite being unable to quantify the compounds, the absolute peak areas were used to establish a hypothesis, based on samples being analyzed in triplicate, using relatively new fibers (i.e., <50 injections), with no sign of degradation. The response of *m*/*z* 124 was generally higher in agitated samples than in non-agitated samples, provided the same volume of internal standard was used. In contrast, the opposite was observed for the response of *m*/*z* 127 (data not shown). To provide an explanation, the auto-sampler’s agitation process was evaluated, and it was found that agitation had a variable effect on both the sample and the internal standard solution. At 250 rpm the agitator moved the headspace vial in a horizontal circular trajectory, with the inserted ampoule spinning within the vial. This caused the ampoule to sit at an angle in the vial, which impacted the relative abundance of *m*/*z* 124 in the headspace of agitated samples. According to Dalton’s Law, the total pressure in a gas phase equals the sum of pressure of each individual component. In the current case, the headspace pressure in the vial is comprised of the vapor pressure of both the sample and the internal standard solution. The distribution of each volatile component is defined by its Henry’s Law constant. In the concept of HS-SPME, Pawliszyn indicated that Henry’s Law constant is only dependent on the system temperature and the liquid phase matrix [31]. Considering the sample vials were left unagitated prior to extraction, it can be concluded that agitation-induced partial pressure differences during extraction disrupted the headspace pressure distribution. However, this disruption doesn’t alter the Henry’s Law constant of any of the volatile compounds, or the final equilibrium, and it becomes less effective as the system approaches equilibrium. As a consequence, samples were extracted without agitation in the final HS-SPME method developed in this study.

#### 2.2.2. Influence of Volume of Internal Standard Solution

According to Henry’s Law Constant, equation K_1_ = C_s_/C_h_ can be rearranged to give: C_h_ = C_0_/(K_1_ + β); where β is the phase ratio between the headspace and the liquid phase of the sample. In the recently updated Henry’s Constant Compilation [32], the value of K for guaiacol partitioning between water and air is around 2.2 × 10^4^ at 25 °C (converted from reported H_cp_ mol/m^3^ Pa). In the current study, the phase ratio (β) between the additional liquid phase and the headspace phase ranged from 6 to 139, which is insignificant when added to K (the volume of the glass material of the ampoule was deemed negligible). It can be inferred that the concentration of internal standard in the headspace at equilibrium would be within similar ranges for the various internal standard volumes used. It was concluded that agitation disrupted the equilibrating process, albeit only small deviations were observed in the RPA of agitated samples when the internal standard volume was 0.1 mL (Figure 2). This indicated that the system approached equilibrium sooner with smaller internal standard volumes. As such, lower volumes of internal standard were used in the new HS-SPME method. Taking into account the possible depletion of the deuterium labels in the internal standard [33], 0.5 mL was chosen as the functional volume for the internal standard.

#### 2.2.3. Influence of Extraction Temperature, Extraction Time, and Internal Standard Concentration

**Figure 3:**
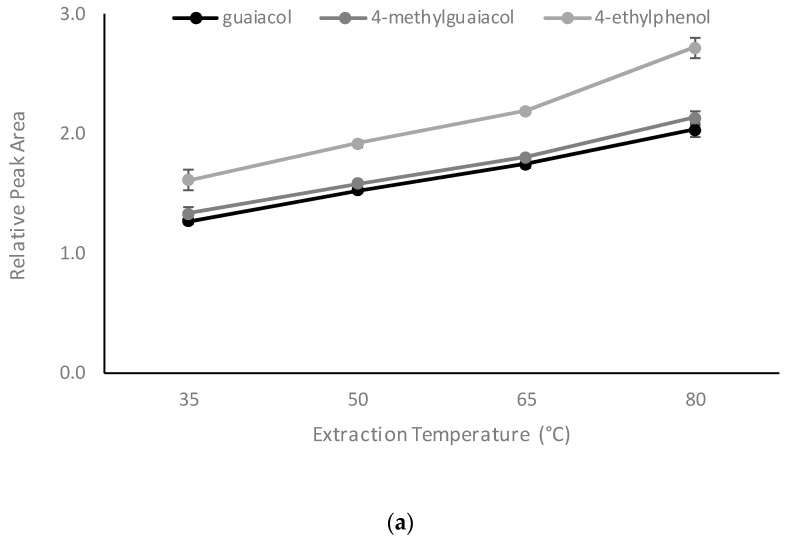

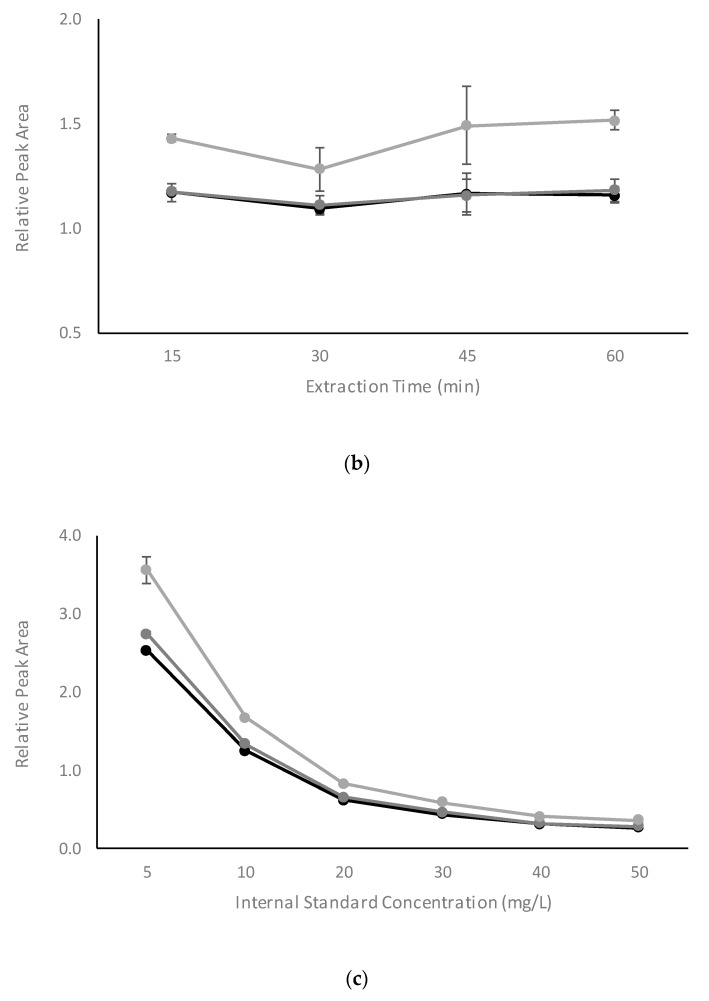
Effect of (**a**) extraction temperature, (**b**) extraction time, and (**c**) internal standard concentration on the relative peak area of guaiacol, 4-methylguaiacol, and 4-ethylphenol. Values are means of three replicates ± standard error (but some standard errors are obscured by symbols).

Once the key analytical parameters had been optimized, several other factors, i.e., extraction temperature, duration, and internal standard concentration, were evaluated. Increasing RPAs were observed for volatile phenols when extraction temperature was increased from 35 to 80 °C (Figure 3). As mentioned above, Henry’s Law constant (K) is temperature dependent, so K decreased as the extraction temperature increased for most volatile phenols [32]. Wieland and colleagues reported a 100-fold decrease in Henry’s law constant for guaiacol, when the temperature increased from 35 to 80 °C [34]. The phase ratio (β) for the internal standard and sample was 27 and 2.25, respectively (for 0.5 mL of internal standard). According to C_h_ = C_0_/(K + β), with decreasing K, C_h_ will have greater increases at low β values. Three things need to be taken into consideration when choosing extraction temperature: experimental sensitivity; the stability of CD complexation; and the potential for volatile compounds present in the headspace to re-dissolve in either of the liquid phases (i.e., the sample or the internal standard solution). In the present study, a temperature of 35 °C was therefore chosen as a trade-off.

Since the equilibrium problem was resolved by avoiding agitation and minimizing the volume of internal standard, optimization of the extraction time largely addressed the analytical sensitivity. The RPA for volatile phenols did not show significant differences between extraction times, ranging from 15 to 60 min (Figure 3). Accordingly, 15 min was chosen as the extraction time for the new HS-SPME method.

In terms of internal standard concentration, according to C_h_ = C_0_/(K + β) and RPA = A_s_/A_i_, it is expected that the RPA will follow a rational function with linear increases in concentration of the internal standard. Experimental data supported this notion (Figure 3). Indeed, in quantification studies using an isotopically labelled standard, the concentration is normally irrelevant to the analysis of compounds of interest, except where the standard is used for calibration [35]. The concern with choosing a higher concentration of ISTD is the adsorption capacity of the SPME fiber and competition for absorption between the standard and the compounds of interest. In the current study, 10 mg/L gave a RPA range close to 1.0.

### 2.3. Experimental Conditions for the Four-Phase HS-SPME GC-MS Method

The final method involved a 6-mL aliquot of model wine solution spiked with volatile phenols (at 1 mg/L) being placed into a 20-mL headspace vial. A 0.5-mL aliquot of internal standard solution (10 mg/L) was added to a 2-mL glass ampoule, which was then inserted into the SPME vial. The vial was incubated for 10 min at 35 °C before extraction with the SPME fiber for 15 min. No agitation was employed during extraction. The new HS-SPME GC MS method gave excellent linearity and repeatability (Table 2). A calibration function was constructed for guaiacol, 4-methylguaiacol, and 4-ethylphenol ranging from 0.25 to 2 mg/L, and also gave excellent linearity, with an R^2^ value ≥ 0.9956.

### 2.4. Retention of Volatile Phenols by α-CD, β-CD, and γ-CD in Model Wine

Different CDs exhibited varying degrees of binding with the volatile phenols studied, i.e., guaiacol, 4-methylguaiacol, 4-ethylphenol, *o*-cresol, *m*-cresol, *p*-cresol, 4-ethylguaiacol, and eugenol (Table 3). In the current study, β-CD retained the highest proportion of volatile phenols, with the overall headspace concentration of volatile phenols reduced to 48.3% following the addition of 25 g/L β-CD. This was not unexpected, given β-CD is the most frequently reported inclusion complex host in other CD studies, because of its cavity size and hydrophobicity [19]. Guaiacol proved to be the most difficult compound to encapsulate within the CDs, with a 25 g/L addition of γ-CD achieving the best removal of guaiacol (being almost 30%). In contrast, 4-ethylphenol was most susceptible to CD complexation, and β-CD reduced the headspace concentration of 4-ethylphenol to just 23.1%. The ranking of volatile phenols by the extent to which their headspace concentration decreased following β-CD addition was: 4-ethylphenol > *p*-cresol > eugenol > *m*-cresol > 4-ethylguaiacol > *o*-cresol > 4-methylguaiacol > guaiacol. Differences in reactivity were attributed to the molecular structure of the volatile phenols. It has long been established that the hydrophobicity, molecular structure, and size of guest molecules are among the most influential factors in the formation of CD inclusion complexes [18,22]. Factors that influence binding between CDs and guest molecules have been previously studied [36]. In the current study, it was obvious that the molecular geometry and the polarity of chemical functional groups in the guest molecule played a major role in binding. The more highly retained 4-ethylphenol and *p*-cresol have the most “aligned” structure, with the non-polar alkyl groups attached in the *para* position of the benzene ring (Figure S2), whereas the less highly retained phenols, namely guaiacol and 4-methylguaiacol, have more polar methoxy groups at their *ortho* positions, which likely act to sterically hinder the molecule from entering into the β-CD cavity.

## 3. Materials and Methods

### 3.1. Chemicals

Analytical grade volatile phenols (guaiacol, 4-methylguaiacol, 4-ethylguaiacol, 4-ethylphenol, *o*-cresol, *m*-cresol, *p*-cresol, and eugenol) and deuterated NMR solvents (*d*_6_-ethanol, D_2_O, and DCl) were purchased from Sigma-Aldrich (Castle Hill, NSW, Australia). Deuterium-labelled internal standards (*d*_3_-guaiacol, *d*_3_-4-methylguaiacol, and *d*_4_-4-ethylphenol) were sourced from CDN Isotopes (Pointe-Claire, Quebec, Canada). Analytical grade ethanol, tartaric acid, and sodium hydroxide were purchased from Thermo Fisher Scientific (Waltham, MA, USA). Food grade (>98% purity) α-, β-, and γ-CDs were supplied by IMCD Group (Adelaide, SA, Australia). Model wine was prepared by dissolving tartaric acid (5 g/L) in aqueous ethanol (12% alcohol by volume) and adjusting the pH to 3.5 by dropwise addition of 1 M sodium hydroxide. Stock solutions of internal standards and volatile phenols were prepared volumetrically in absolute ethanol and stored at −20 °C, with working solutions prepared in model wine and stored at 4 °C.

### 3.2. Nuclear Magnetic Resonance Analysis

Complexation of volatile phenols by CDs was investigated by 2-dimensional nuclear magnetic resonance rotating frame Overhauser effect spectroscopy (^1^H 2D ROESY). Samples were prepared by adding volatile phenols (10^−3^ mol/L) and CDs (10^−2^ mol/L) to deuterated model wine (i.e., 12% *d_5_*-ethanol in D_2_O, pD adjusted to 3.5 by dropwise addition of DCl). Spectra were recorded with an Agilent DD2 600 MHz spectrometer fitted with a cryoprobe (Agilent Technologies, Santa Clara, CA, USA) operating at 600 MHz with a delay time of 300 ms.

### 3.3. Sensory Analysis

Triangle tests [37] were performed to demonstrate the sensory impact of volatile phenol retention by β-CD. The panel comprised 38 postgraduate Wine Business students (8 male and 30 female, aged between 21 and 50 years) from the University of Adelaide. Model wines were presented in three-digit coded, covered XL5 wine glasses, using a balanced, randomized presentation order comprising all possible configurations, i.e., ABB, ABA, AAB, BAA, BAB, and BBA, where A denotes model wine spiked with volatile phenols and B denotes model wine spiked with volatile phenols and treated with β-CD (10 g/L). Panelists evaluated two brackets of wines: one representing smoke taint, comprising model wines spiked with guaiacol, 4-methylguaiacol, and *p*-cresol (1 mg/L each); and one representing *Brettanomyces* spoilage, comprising model wines spiked with 4-ethylphenol and 4-ethylguaiacol (1 mg/L each). Panelists smelled but did not taste wines, then identified the sample in each bracket that was considered to be different.

### 3.4. GC-MS Instrumental Analysis

Analysis of samples was performed with an Agilent GC-MS system (Santa Clara, CA, USA) comprising a 7890A gas chromatograph equipped with a Gerstel MPS autosampler (Mülheim, Germany) coupled to a 5975C mass selective detector. A DB-Wax column (60 m, 0.25 mm id, 0.25 µm film thickness, Agilent J&W, Folsom, CA, USA) was used for separation. The carrier gas was helium (BOC Gas, Adelaide, SA, Australia), at a constant flow of 1.5 mL/min. The inlet temperature was set at 240 °C and the oven temperature started at 40 °C for 1 min, increased to 200 °C at 5 °C/min, and was held at 200 °C for 5 min, before being increased to 250 °C at 10 °C/min and remaining at 250 °C for 10 min, giving a total run time of 52 min. The transfer line was set at 230 °C and positive ion electron impact spectra at 70 eV were recorded in the range *m*/*z* 25 to 215 for scan runs. For quantification of volatile phenols, mass spectra were recorded in selected ion monitoring (SIM) mode. The ions monitored in SIM mode were: *m*/*z* 109, *124* for guaiacol; *m*/*z* 109, *127* for *d_3_*-guaiacol; *m*/*z* 123, *138* for 4-methylguaiacol; *m*/*z* 126, *141* for *d_3_*-4-methylguaiacol; *m*/*z* 77, 90, *108* for *o*-cresol; *m*/*z* 122, 137, *152* for 4-ethylguaiacol; *m*/*z* 77, *107* for *p*-cresol; *m*/*z* 79, *108* for *m*-cresol; *m*/*z* 77, *122* for 4-ethylphenol; *m*/*z* 77, *126* for *d_4_*-4-ethylphenol; and *m*/*z* 149, *164* for eugenol; with italicized ions used for quantitation. Volatile phenol concentrations are reported as relative peak areas (RPA), i.e., as the ratio of the peak area of the analyte (A_s_) relative to the peak area of the isotopic standard (A_i_).

### 3.5. HS-SPME GC-MS Analysis of Volatile Phenols in Model Wine Following CD Addition

An HS-SPME GC-MS method developed by other studies for determination of volatile phenols in wine (Castro-Mejías et al. 2003) was initially employed in the current study to determine the changes in volatile phenol levels following CD addition in model wine. Model wine was spiked with guaiacol, 4-methylguaiacol, or 4-ethylphenol at 0, 0.25, 0.5, 0.75, 1.0, 1.25, 1.5, 1.75, and 2 mg/L, and an aliquot of normalizing internal standard solution (containing 100 mg/L each of *d_3_*-guaiacol, *d_3_*-4-methylguaiacol, and *d_4_*-4-ethylphenol) added, prior to SPME GC-MS analysis to develop calibration functions (Table S1). High linearity was observed over the working range, with correlation coefficients greater than 0.9995. A preliminary experiment using the above SPME GC-MS method involving the addition of α-CD, β-CD, or γ-CD (of 25 g/L) to model wine solutions containing guaiacol, 4-methylguaiacol, and 4-ethylphenol (1 mg/L each) suggested no significant binding of volatile phenols by the CDs; i.e., no significant differences were observed between the RPAs of compound-to-standard for volatile phenols with and without CD addition (Table 1). However, the absolute peak areas of analytes (and internal standards) were observed to be considerably smaller in samples with CD addition, e.g., the peak areas of 4-ethylphenol and *d_4_*-4-ethylphenol reduced from approximately 100,000 to 20,000 abundance. Initially this was thought to reflect either variation in fiber performance or fiber degradation, but subsequent sensory and NMR analyses (described below) confirmed CD binding of volatile phenols, which led to the conclusion that CDs were also binding to the isotopically labelled standards and prompted the development of a novel HS-SPME method, involving introduction of the internal standard solution via an additional liquid phase, so as to prevent inclusion of standards by CDs. This was achieved by inserting a 2-mL glass ampoule (Gerresheimer Shuangfang Pharmaceutical Packaging, Zhenjiang, China) containing the internal standard solution into the 20-mL headspace vial (Sigma Aldrich, Castle Hill, NSW, Australia), as shown in Figure S3. A series of experiments (using a solution of methylene blue) were performed to ensure there was no mixing of samples in the SPME vial and the glass ampoule (or vice-versa), during sample preparation, or transfer, agitation, and extraction (data not shown). The influence of internal standard volume, agitation, incubation (temperature and duration), and the duration of sample extraction on the repeatability and accuracy of the novel SPME method were also evaluated, as method development and validation.

### 3.6. Method Development for the Four-Phase HS-SPME GC-MS Method

#### 3.6.1. Influence of Agitation, Internal Standard Volume, and Pre-Analysis Equilibration Time

An aliquot of model wine (6 mL) containing 1 mg/L of guaiacol, 4-methylguaiacol, and 4-ethylphenol was transferred into headspace vials. This volume maximized the sample being analyzed, while ensuring the ampoule remained submerged. The inserted normalizing standard solution contained three isotopic standards (*d_3_*-guaiacol, *d_3_*-4-methylguaiacol, and *d_4_*-4-ethylphenol) at 10 mg/L each. In preliminary benchtop experiments, both agitation and the volume of inserted liquid were found to change during the pre-analysis equilibration time (data not shown). Therefore, a multiple factorial design was adopted to optimize the extraction conditions (performed in triplicate). Four different volumes of internal standard were used (0.1, 0.5, 1.0, and 2.0 mL). Samples were analyzed over a 24-h period (at 3-hr intervals, in triplicate) to determine the optimal equilibration time. Agitation, when used, was set at 250 rpm. The autosampler incubation and extraction times were 10 and 15 min, respectively, and the extraction temperature was 35 °C.

#### 3.6.2. Influence of Extraction Time, Extraction Temperature, and Internal Standard Concentration

Using the optimized parameters identified above, several additional parameters were evaluated. Extraction temperatures of 35, 50, 65, and 80 °C, extraction times of 15, 30, 45, and 60 min, and internal standard concentrations of 5, 10, 20, 30, 40, and 50 mg/L were evaluated; with all samples prepared in triplicate.

### 3.7. Method Performance for the Four-Phase HS-SPME GC-MS Method

The optimized SPME method comprised the following conditions: an ampoule tube containing 0.5 mL of model wine spiked with 10 mg/L of internal standard solution was inserted into 6 mL of sample (i.e., model wine spiked with volatile phenols) in a 20-mL headspace sampling vial. Equilibrium in the headspace vial was achieved via 15-min incubation at 35 °C, before 15-min extraction without agitation. To validate the method, calibration curves were generated for guaiacol, 4-methylguaiacol, 4-ethylguaiacol, 4-ethylphenol, *o*-cresol, *m*-cresol, *p*-cresol, and eugenol, spiked at 0, 0.25, 0.5, 0.75, 1.0, 1.25, 1.5, 1.75, and 2.0 mg/L. Guaiacol, 4-methylguaiacol, and 4-ethylphenol were quantified against their isotopically labelled equivalents, whereas 4-ethylguaiacol, *o*-cresol, *m*-cresol, *p*-cresol, and eugenol were quantified against *d_3_*-guaiacol. The linear range of detection was subsequently validated at volatile phenol concentrations up to 50 mg/L. Samples spiked with 0.25, 0.5, 1.0, 1.25, 1.75, or 2.0 mg/L of guaiacol, 4-methylguaiacol, and 4-ethylphenol were prepared in triplicate and analyzed to evaluate method repeatability; with preparation and analysis of a subset of samples (i.e., samples spiked at 0.5, 1.25, and 1.75 mg/L) repeated after 1 month. All validation samples were analyzed in triplicate.

### 3.8. Retention of Volatile Phenols in Model Wine by Cyclodextrins

A model wine solution comprising 1 mg/L of guaiacol, 4-methylguaiacol, 4-ethylguaiacol, 4-ethylphenol, *o*-cresol, *m*-cresol, *p*-cresol, and eugenol was prepared. Aliquots (6 mL) were placed in 20-mL SPME headspace vials, to which 25 g/L of α-CD, β-CD, or γ-CD were added. Samples were then heated to 35 °C in an incubator (Ratek, Boronia, VIC, Australia) with agitation (200 rpm) for 20 min, after which samples were cooled to ambient temperature and analyzed by GC-MS. Samples were prepared in triplicate. Control samples (i.e., samples without the addition of CD) were also prepared in triplicate. The residual volatile phenol levels were determined using the optimized four-phase SPME GC-MS method. Semi-quantification based on standard addition was used to calculate the percentage difference between the RPA of residual volatile phenols following CD addition, with those of control samples.

### 3.9. Data Analysis

Data are presented as mean values of three replicates ± standard error. One-way ANOVA was conducted to determine the differences between sample means, with a T-test at *p* = 0.05, using XLSTAT software (version 2015.3, Addinsoft, Paris, France).

## 4. Conclusions

The newly developed four-phase HS-SPME GC-MS method overcame the difficulties associated with CD encapsulation of internal standards spiked directly into samples. Although this method does not completely prevent interactions between isotopically labelled standards and dissolved CDs, it mitigates interactions by introducing the standard via a separate liquid phase. Modification and validation therefore need to be undertaken when adapting this method for the analysis of other volatile compounds and in more complicated substrates (e.g., wine, rather than model wine). The improvements offered by this method nevertheless enabled complexation between CDs and volatile phenols to be studied. CDs were found to form inclusion complexes with volatile phenols in model wine, resulting in reductions in the perceived intensity of off-odors. Importantly, this method can be adapted for quantitative analysis of other systems in which a substrate component might similarly scavenge internal standards.

## Figures and Tables

**Table 1:** Residual volatile phenol levels (as relative peak area) following addition of α-, β-, and γ-CDs to model wine, based on conventional three-phase headspace solid phase microextraction (HS-SPME) gas chromatography-mass spectrometry (GC-MS) analysis.

	Guaiacol	4-Methylguaiacol	4-Ethylphenol
Control	0.99 ± 0.02	1.00 ± 0.01	1.22 ± 0.02
α-CD	0.99 ± 0.02 (100%)	1.00 ± 0.02 (100%)	1.23 ± 0.02 (100.8%)
β-CD	0.99 ± 0.01 (100%)	1.00 ± 0.01 (100%)	1.29 ± 0.02 (105.7%)
γ-CD	0.99 ± 0.02 (100%)	1.00 ± 0.02 (100%)	1.24 ± 0.03 (101.6%)

Values are means of three replicates ± standard error (and percentage of control). Values within columns were not significantly different (one-way ANOVA, *p* = 0.05).

**Table 2:** Validation of the four-phase HS-SPME GC-MS method.

	Guaiacol		4-Methylguaiacol		4-Ethylphenol	
	RPA	CV (%)	RPA	CV (%)	RPA	CV (%)
0.25 mg/L	0.35	2.7	0.38	2.5	0.48	1.7
0.5 mg/L	0.63 (0.65)	3.1 (4.5)	0.68 (0.69)	2.2 (5.8)	0.86 (0.90)	5.4 (8.9)
1.0 mg/L	1.12	7.5	1.16	8.9	1.50	9.3
1.25 mg/L	1.61 (1.53)	0.6 (5.4)	1.74 (1.58)	0.8 (5.8)	2.27 (1.99)	6.5 (7.0)
1.75 mg/L	2.12 (2.10)	0.1 (2.2)	2.27 (2.17)	0.2 (3.1)	3.01 (2.84)	6.6 (6.1)
2.0 mg/L	2.31	0.7	2.36	0.8	2.97	2.4

Values are means of three replicates (and repeat analyses performed after 1 month). RPA = relative peak area; CV = coefficient of variation.

**Table 3:** Residual volatile phenol levels following addition of α-, β-, and γ-CDs to model wine, using four-phase HS-SPME GC-MS analysis.

		Guaiacol	4-Methylguaiacol	4-Ethylphenol	4-Ethylguaiacol	*o*-Cresol	*m*-Cresol	*p*-Cresol	Eugenol
Control		1.26 a ± 0.05	1.36 a ± 0.06	1.85 a ± 0.10	0.94 a ± 0.01	1.64 a ± 0.11	0.95 a ± 0.05	1.86 a ± 0.06	0.66 a ± 0.03
α-CD	5 g/L	1.14 abc ± 0.02	1.19 ab ± 0.04	1.51 ab ± 0.10	0.82 b ± 0.01	1.39 abc ± 0.06	0.81 b ± 0.02	0.92 b ± 0.03	0.59 ab ± 0.03
		(90.3%)	(87.9%)	(81.6%)	(87.3%)	(85.0%)	(85.1%)	(84.8%)	(71.1%)
	25 g/L	1.17 ab ± 0.05	1.20 ab ± 0.04	1.42 b ± 0.11	0.76 b ± 0.02	1.41 ab ± 0.07	0.75 bc ± 0.03	0.85 bc ± 0.04	0.47 c ± 0.01
		(92.9%)	(88.2%)	(76.9%)	(80.4%)	(86.2%)	(79.3%)	(78.3%)	(71.1%)
β-CD	5 g/L	1.01 cde ± 0.03	1.04 bcd ± 0.04	0.80 c ± 0.03	0.68 c ± 0.02	1.14 cd ± 0.03	0.61 d ± 0.01	0.61 d ± 0.01	0.46 c ± 0.01
		(80.2%)	(76.7%)	(43.1%)	(72.6%)	(70.0%)	(63.9%)	(56.5%)	(70.0%)
	25 g/L	0.98 de ± 0.00	0.95 cd ± 0.02	0.43 d ± 0.02	0.47 e ± 0.01	0.92 d ± 0.01	0.41 e ± 0.01	0.34 e ± 0.01	0.24 e ± 0.01
		(77.5%)	(69.8%)	(23.1%)	(50.0%)	(56.0%)	(42.6%)	(31.1%)	(36.9%)
γ-CD	5 g/L	1.07 bcd ± 0.01	1.10 bc ± 0.01	1.29 b ± 0.01	0.79 b ± 0.00	1.24 bc ± 0.01	0.67 cd ± 0.01	0.76 c ± 0.01	0.56 b ± 0.01
		(84.8%)	(81.1%)	(69.8%)	(84.2%)	(75.7%)	(70.1%)	(70.0%)	(84.7%)
	25 g/L	0.89 e ± 0.02	0.87 d ± 0.03	0.77 cd ± 0.03	0.57 d ± 0.01	0.93 d ± 0.01	0.49 e ± 0.01	0.56 d ± 0.01	0.35 d ± 0.01
		(70.6%)	(64.1%)	(41.5%)	(60.0%)	(57.2%)	(51.1%)	(51.5%)	(53.0%)

Values are means of three replicates ± standard error (and percentage of control). Values followed by different letters within columns are statistically significant (one-way ANOVA, *p* = 0.05).

## Supplementary Materials

The following are available online at https://www.mdpi.com/1420-3049/24/19/3432/s1, Table S1: Calibration curve using conventional HS-SPME GC-MS method. Figure S1: Structures of α-CD, β-CD, and γ-CD. Figure S2: Encapsulation of 4-ethylphenol within β-CD. Figure S3: Diagram of headspace vial containing model wine sample, with different volumes of internal standard in the glass ampoule (as indicated by shading).
