# Supplementary material for: Development and Evaluation of a HS-SPME GC-MS Method for Determining the Retention of Volatile Phenols by Cyclodextrin in Model Wine
Source: Molecules. 2019 Sep 21;24(19):3432. doi: 10.3390/molecules24193432 (PMC6803902; doi:10.3390/molecules24193432)
Supplement: Supplementary file 1 [file molecules-24-03432-s001.pdf]

Supplementary Materials

Development and Evaluation of a Novel SPME GC-MS Method for Determining the Retention of Volatile Phenols by Cyclodextrin in Model Wine

Chao Dang [1,2], Kerry L. Wilkinson [1,2,*], Vladimir Jiranek [1,2] and Dennis K. Taylor [1,2]

[1] The University of Adelaide, School of Agriculture, Food and Wine, PMB 1, Glen Osmond, SA, 5064, Australia; chao.dang@adelaide.edu.au (C.D.), vladimir.jiranek@adelaide.edu.au (V.J.), dennis.taylor@adelaide.edu.au (D.K.T.)

[2] The Australian Research Council Training Centre for Innovative Wine Production, PMB 1, Glen Osmond, SA, 5064, Australia

* Correspondence: kerry.wilkinson@adelaide.edu.au (K.L.W.); Tel.: +61-8-8313-7360

Table S1. Calibration curve using conventional HS-SPME GC-MS method.

	**Guaiacol**	**4-Methylguaiacol**	**4-Ethylguaiacol**
Slope	0.980	1.014	1.328
Intercept	0.003	0.007	0.006
R^2	0.9997	0.9996	0.9996

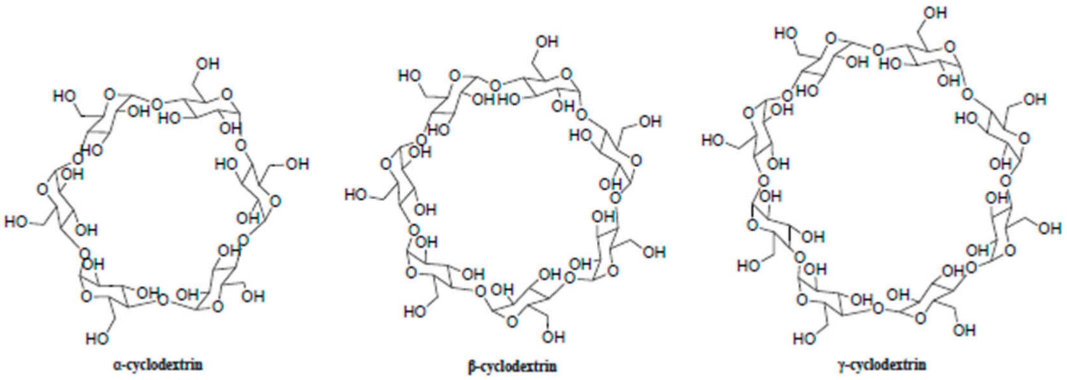

Figure S1. Structures of *α*-CD, *β*-CD and *γ*-CD.

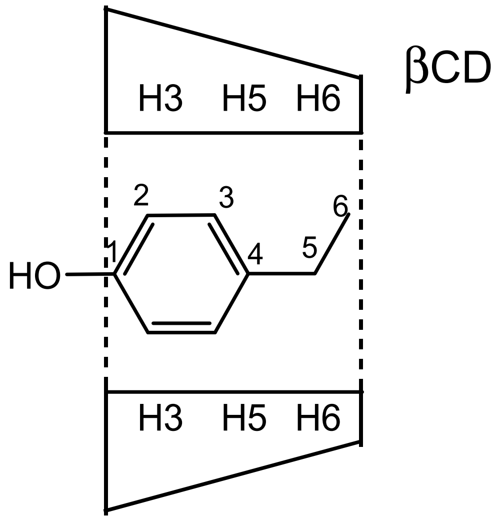

Figure S2. Encapsulation of 4-ethylphenol within *β*-CD.

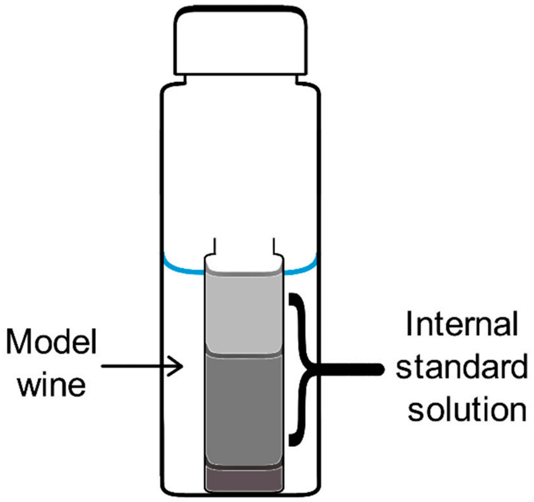

Figure S3. Diagram of headspace vial containing model wine sample, with different volumes of internal standard in the glass ampoule (as indicated by shading).
